# Editorial: Reconstruction of the Foot and Ankle

**DOI:** 10.2174/1874325001711010609

**Published:** 2017-07-31

**Authors:** Tun Hing Lui

**Affiliations:** 1 North District Hospital, Department of Orthopaedics and Traumatology, 9 Po Kin Road, Sheung Shui, Hong Kong

Reconstruction of the foot and ankle is an increasingly popular topic with multiple advances and breakthroughs. Improved surgical methods, and better implants have raised the expectation of both patients and surgeons alike; and the principle of re-creating a stable, shoeable, plantigrade foot has shifted from being the 'ultimate aim' to more of a 'minimal requirement'. Currently, there are a myriad of different osteotomies, tendon transfers *etc* that are commonly used worldwide. It is often stated that if there is a multitude of management choices for similar pathologies, it usually indicates that no specific strategy or treatment option is significantly superior than the others. Many of the more complex reconstructions are performed on an a la carte basis, requiring careful calibration in the axial, sagittal and coronal planes. Once we factor in the meticulous soft tissue balancing required in dynamic reconstructions; it is not hard to fathom the complexity of the surgical plans. The topic of Foot and ankle reconstruction is currently undergoing a phase of rapid development and cultivation. It can be anticipated that if this trend of investigation and publishing continues in this realm; our breadth of knowledge will expand exponentially and we will have an increasingly useful armamentarium of surgical methods to tackle foot deformities. In this thematic issue; we have compiled a list of interesting articles elucidating different aspects of this large topic of foot and ankle reconstruction.

